# Bioenergy production data from anaerobic digestion of thermally hydrolyzed organic fraction of municipal solid waste

**DOI:** 10.1016/j.dib.2019.01.018

**Published:** 2019-01-14

**Authors:** A.S. Razavi, E. Hosseini Koupaie, A. Azizi, H. Hafez, E. Elbeshbishy

**Affiliations:** a Environmental Research Group for Resource Recovery, Civil Engineering Department, Faculty of Engineering, Architecture and Science, Ryerson University 350 Victoria Street, Toronto, ON, Canada M5B 2K3; b Greenfield Global, Chatham, ON, Canada N7M 5J4

## Abstract

The presented dataset in this data article provides quantitative data on the production of bioenergy (biogas and biomethane) from mesophilic batch anaerobic digestion (AD) of thermally hydrolyzed organic fraction of municipal solid waste (OFMSW). The discussion and interpretation of the data are provided in another publication entitled “Hydrothermal Pretreatment of Source Separated Organics for Enhanced Solubilization and Biomethane Recovery” (Razavi et al., 2019). The data and information presented in the current data article include (1) the ratio of soluble to particulate chemical oxygen demand (COD) under different thermal hydrolysis condition, (2) the daily measured biogas and biomethane data, (3) the cumulative methane yield data in terms of mL CH_4_ produced per gram of volatile suspended solids (VSS) as well as feedstock added, (4) the ultimate methane yield data as well as the relative improvement in methane recovery compared to the control (non-hydrolyzed) digester, (5) the data of first-order organics biodegradation rate constants, (6) the procedure of measuring biogas composition via gas chromatography, (7) the procedure of converting the biogas/methane volume data acquired under the actual experimental condition (mesophilic temperature of 38 °C and atmospheric pressure) to the standard temperature (0 °C) and pressure (1 atm) condition, and (8) the procedure of determining the first-order kinetic rate constants.

**Specifications table**

**Table:** 

Subject area	Environmental engineering
More specific subject area	Anaerobic digestion, biological treatment, thermal hydrolysis, waste minimization, bioenergy
Type of data	Table, figures
How data were acquired	The gas chromatography was employed to determine the methane content of the produced biogas. A Hach spectrophotometer (model DR3900) was used the analysis the chemical oxygen demand (COD) of the samples calorimetrically. The volume of the produced biogas was measured manually with a Poulten & Graf Fortuna™ air-sealed glass syringe (capacity of 100 m) throughout the biochemical methane potential (BMP) assay. To analyze the soluble COD (SCOD), the samples were centrifuged for 20 min at 10,000 rpm using a Sorvall Legend XT centrifuge (Fisher Scientific, US). Then, the liquid fraction (supernatant) of the centrifuged samples was passed through 0.45 µm microfiber filters. The analysis including the analysis of ANOVA and the interactions analysis was done using Minitab Software 17.
Data format	Raw, analyzed
Experimental factors	Thermal hydrolysis parameters include temperature (°C), holding time (min), pressure (kPa), and severity index (–). All the digesters were operated at the mesophilic temperature of 38 °C.
Experimental features	Thermal hydrolysis experiments were conducted under wide ranges of temperature, retention time, and pressure so that it covers the severity index range of 3–5 commonly used in industrial applications. Fifteen different thermal hydrolysis conditions were applied to the OFMSW samples. The thermal hydrolysis temperature, pressure, and holding time ranged from 150 to 240 °C, 476 to 3367 kPa, and 5 to 30 min, respectively. The BMP test was performed using raw (non-pretreated) and thermally hydrolyzed OFMSW samples. The BMP assay as well as the sample analyses were performed in triplicates.
Data source location	Toronto, Canada
Data accessibility	Data are presented in this article
Related research article	A.S. Razavi, E. Hosseini Koupaie, A. Azizi, H. Hafez, E. Elbeshbishy, Hydrothermal pretreatment of source separated organics for enhanced solubilization and biomethane recovery, Bioresour. Technol. [1]

**Value of the data**

• The data explain the procedure for converting the gas volume data obtained under specific experimental conditions (e.g., specific temperature and/or pressure) into the values under a standard condition (e.g. 0 °C, 1 atm).
• Data standardization provide the opportunity to compare the data acquired under different experimental conditions.
• The dataset covering a wide range of thermal hydrolysis conditions might be used as a benchmark to validate the findings of other studies.
• The data highlight the importance of selecting the optimum ranges of temperature, pressure, and retention time for thermal hydrolysis of OFMSW prior to the AD process.
• The kinetics rate data provide valuable information regarding the rate of the anaerobic digestion thermally hydrolyzed OFMSW.

## 1 Data

**Fig. 1:**
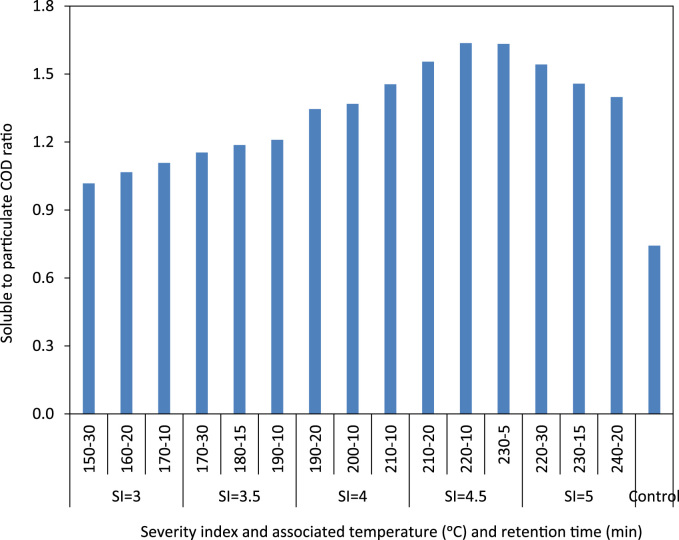
The ratio of soluble to particulate COD.

**Fig. 2:**
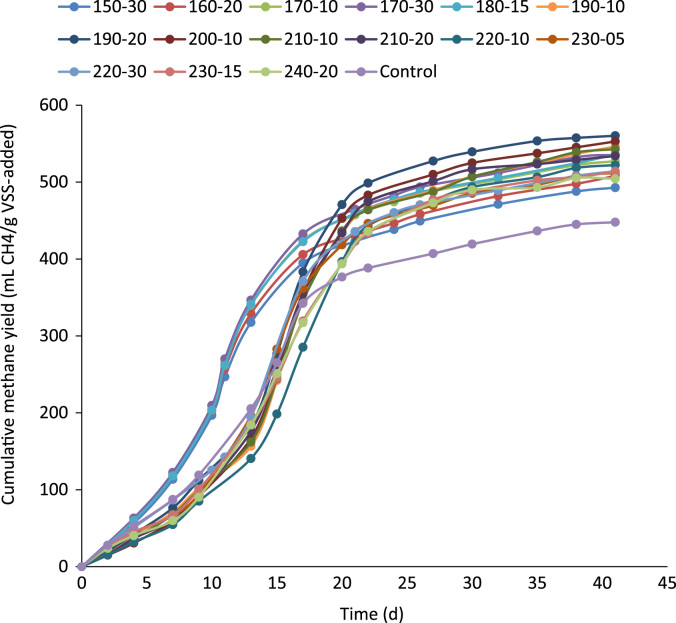
The cumulative methane yields of the BMP digesters as mL CH_4_/g VSS-added.

**Fig. 3:**
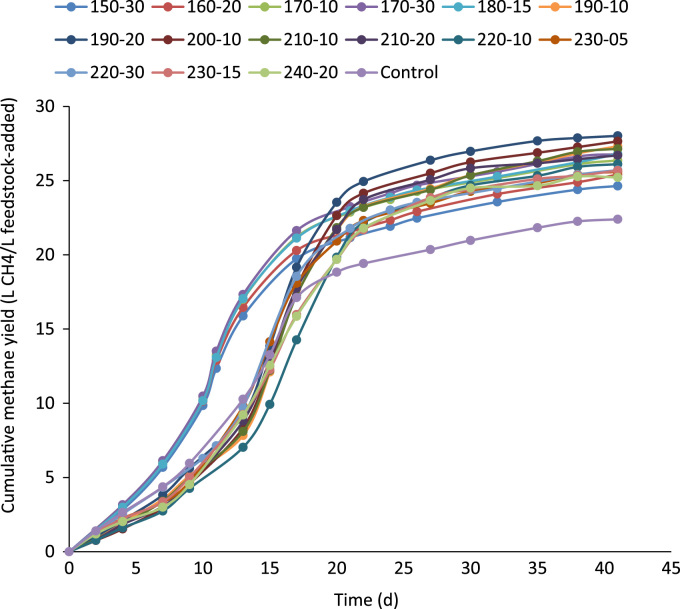
The cumulative methane yields of the BMP digesters as L CH_4_/L feedstock-added.

**Fig. 4:**
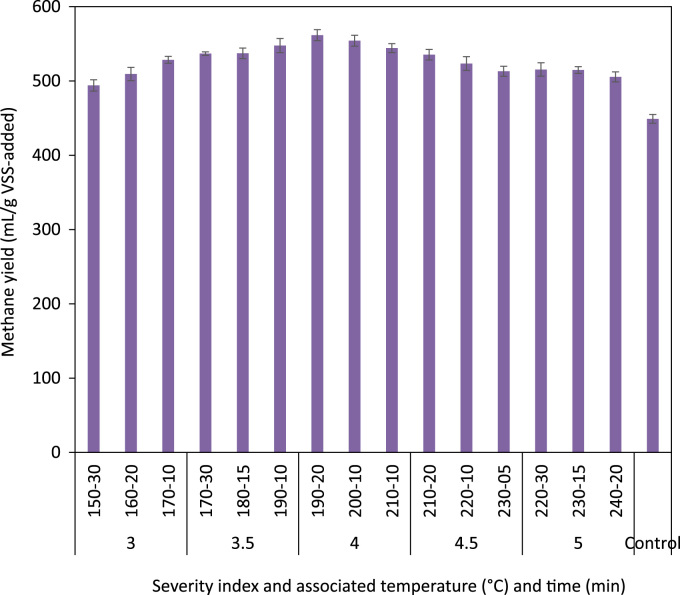
The ultimate methane yield as mL CH4/g VSS-added.

**Fig. 5:**
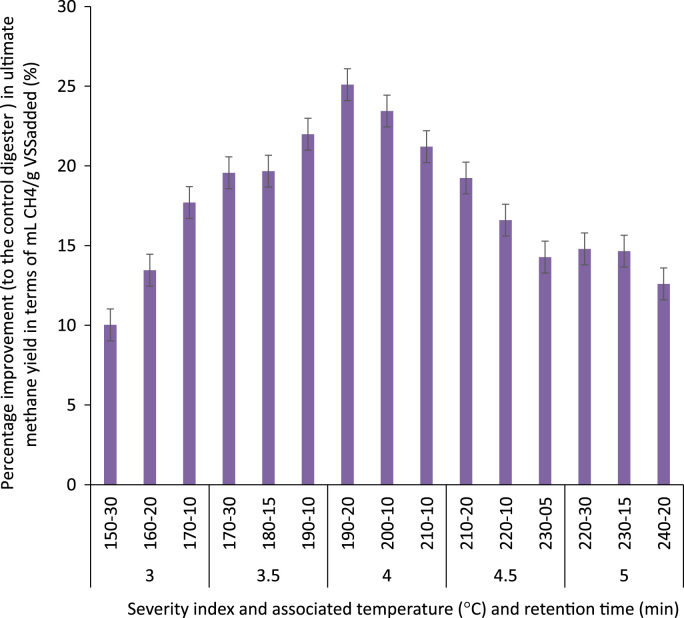
Percentage improvement in ultimate methane yield compared to the control (non-pretreated) digester (%).

**Fig. 6:**
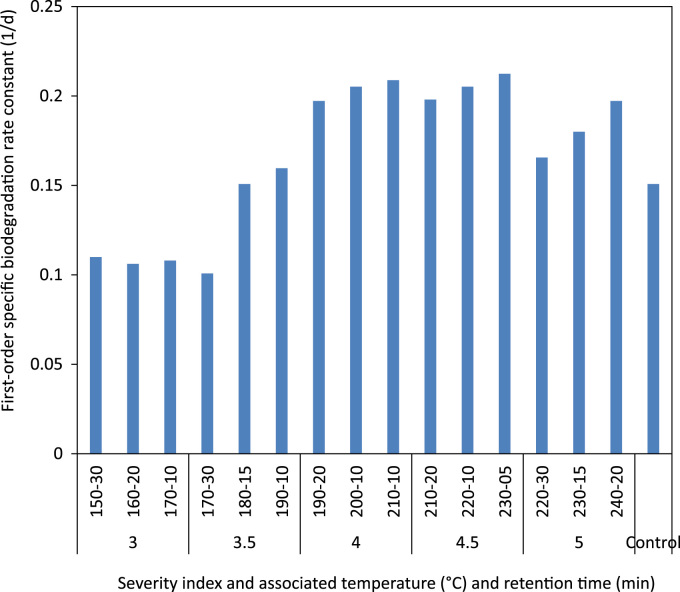
First-order specific biodegradation rate constant of the raw and thermally hydrolyzed digesters.

The ratio of soluble to particulate COD in the raw and thermally hydrolyzed OFMSW samples are compared in Fig. 1. The experimentally measured biogas and biomethane production data throughout the BMP experiment are presented in Tables 1 and 2, respectively. The cumulative biomethane yield in terms of mL CH4/g VSS-added and L CH4/L feedstock-added are illustrated in Figs. 2 and 3, respectively. The ultimate methane yield of the digesters fed with raw and thermally hydrolyzed substrates are compared in Fig. 4. The percentage improvements in the ultimate methane yield of the thermally hydrolyzed digesters in comparison with that of the control digester are shown in Fig. 5. The first-order specific biodegradation rate constants of the BMP digesters are presented in Fig. 6.

**Table 1:** Daily biogas production data from the BMP digesters at a temperature of 38 °C (mL).

**Time (day)**	**Severity index (SI) temperature (°C)-holding time (min)**															**Control**
	**3**			**3.5**			**4**			**4.5**			**5**			
	**150** **°C 30** **min**	**160** **°C 20** **min**	**170** **°C 10** **min**	**170** **°C 30** **min**	**180** **°C 15** **min**	**190** **°C 10** **min**	**190** **°C 20** **min**	**200** **°C 10** **min**	**210** **°C 10** **min**	**210** **°C 20** **min**	**220** **°C 10** **min**	**230** **°C 05** **min**	**220** **°C 30** **min**	**230** °**C 15** **min**	**240** **°C 20** **min**	
0	0	0	0	0	0	0	0	0	0	0	0	0	0	0	0	0
2	42	54	43	59	47	31	36	26	30	28	27	43	37	45	42	49
4	99	110	111	111	105	68	74	54	63	65	56	79	89	78	71	93
7	199	209	210	215	207	124	134	104	119	107	96	116	152	118	105	153
9	345	355	359	367	356	179	197	160	175	164	149	166	220	176	158	209
13	433	451	464	473	459	274	321	292	284	309	246	344	250	324	323	360
15	557	577	596	607	598	424	485	439	428	453	348	496	340	430	440	465
17	692	711	743	758	740	620	672	624	606	622	500	634	650	560	556	600
20	741	755	802	815	805	760	825	794	765	761	695	733	763	690	690	660
22	768	783	831	846	837	815	874	846	813	831	777	782	806	760	764	680
27	787	803	851	865	854	857	924	894	854	878	835	823	824	836	829	713
30	826	844	882	897	886	889	945	920	889	906	865	851	857	856	859	735
35	855	873	915	933	920	920	970	942	922	917	888	871	890	880	864	765
38	860	885	919	936	924	941	977	956	944	927	909	891	895	888	886	780
41	864	891	924	939	939	958	982	969	951	936	915	897	901	900	884	785

**Table 2:** Daily biomethane production data from the BMP digesters at a temperature of 38 °C (mL).

**Time (day)**	**Severity index (SI) temperature (°C)-holding time (min)**															**Control**
	**3**			**3.5**			**4**			**4.5**			**5**			
	**150** **°C 30** **min**	**160** **°C 20** **min**	**170** **°C 10** **min**	**170** **°C 30** **min**	**180** **°C 15** **min**	**190** **°C 10** **min**	**190** **°C 20** **min**	**200** **°C 10** **min**	**210** **°C 10** **min**	**210** **°C 20** **min**	**220** **°C 10** **min**	**230** **°C 05** **min**	**220** **°C 30** **min**	**230** **°C 15** **min**	**240** **°C 20** **min**	
0	0	0	0	0	0	0	0	0	0	0	0	0	0	0	0	0
2	27	35	28	38	31	20	23	17	20	18	18	28	24	29	27	32
4	64	72	72	72	68	44	48	35	41	42	36	51	58	51	46	60
7	129	136	137	140	135	81	87	68	77	70	62	75	99	77	68	99
9	224	231	233	239	231	116	128	104	114	107	97	108	143	114	103	136
13	281	293	302	307	298	178	209	190	185	201	160	224	163	211	210	234
15	362	375	387	395	389	276	315	285	278	294	226	322	221	280	286	302
17	450	462	483	493	481	403	437	406	394	404	325	412	423	364	361	390
20	482	491	521	530	523	494	536	516	497	495	452	476	496	449	449	429
22	499	509	540	550	544	530	568	550	528	540	505	508	524	494	497	442
27	512	522	553	562	555	557	601	581	555	571	543	535	536	543	539	463
30	537	549	573	583	576	578	614	598	578	589	562	553	557	556	558	478
35	556	567	595	606	598	598	631	612	599	596	577	566	579	572	562	497
38	559	575	597	608	601	612	635	621	614	603	591	579	582	577	576	507
41	562	579	601	610	610	623	638	630	618	608	595	583	586	585	575	510

## 2 Experimental design, materials and methods

### 2.1 Procedure of volume data conversion to the standard temperature & pressure condition

The volume of the produced biogas/methane throughout the BMP assay under the mesophilic temperature of 38 °C and atmospheric room pressure was converted to the standard temperature & pressure condition (0 °C and 1 atm) using Eq. (1)

(1) $$V_{\mathrm{STP}}=V_{m}(\frac{P_{m}}{P_{\mathrm{STP}}})(\frac{T_{\mathrm{STP}}}{T_{m}})$$

where,

$V_{\mathrm{STP}}$: Biogas/methane volume of the at the standard temperature & pressure condition (mL)
$V_{m}$: Actual recorded biogas/methane volume (mL)
$P_{m}$: Actual atmospheric pressure at the time of recording the biogas/methane volume (atm)
$P_{\mathrm{STP}}$: Standard pressure (1 atm)
$T_{\mathrm{STP}}$: Standard temperature (273.15°C)
$T_{m}$: Digester temperature (273.15+38=311.15°C)

### 2.2 Biodegradation kinetics rate calculation

The data regarding the rate of organics (COD, VSS, etc.) biodegradation through the digestion process were defined by the first-order reaction model [2], [3], [4], [5], [6], [7]. Eq. (2) shows the kinetic reaction model used to calculate the first-order rate constants data for the TCOD degradation of the digesters.

(2) $$r=\frac{\mathrm{dA}}{\mathrm{dt}}=-kA_{t}$$

in which r, k, and $A_{t}$ are respectively the organics removal rate (e.g., TCOD degradation rate in mg/L.d), the first-order specific biodegradation rate constant (1/d), and the remaining concentration of organics (e.g., TCOD concentration in mg/L) at time t. By integrating and rearranging Eqs. (2) and (3) will be obtained as follows:

(3) $$A_{t}=A_{u}e^{-\mathrm{kt}}$$

in which $A_{u}$ is the ultimate biodegradable organics concentration (mg/L), and the rest of the parameters are as defined before.

### 2.3 Analytical procedure

The amount of the daily biogas production was measured manually using a 100 mL air-tight Poulten & Graf Fortuna™ glass syringe. The composition of the biogas produced throughout the BMP assay was measured in terms of CH_4_, CO_2_, and H_2_ gases using a gas chromatograph (Thermo Scientific Trace 1310). The Trace 1310 gas chromatograph was equipped with a packed column (model: TG-Bond Msieve 5A) with 30 m length and diameter of 0.53 mm. It was also utilized a thermal conductivity detector with oven, filament, and detector temperatures of 80, 250, and 100 °C, respectively. The analysis of COD was performed calorimetrically following the closed reflux methodology outlined by the Standard Methods [8]. A Hach spectrophotometer (model DR3900) was used for COD analysis and the measurements were done at the wavelength at 600 nm. The statistical analysis was performed using Minitab Software 17.
