# Supplementary material for: Bioenergy production data from anaerobic digestion of thermally hydrolyzed organic fraction of municipal solid waste
Source: Data Brief. 2019 Jan 14;22:1018–26. doi: 10.1016/j.dib.2019.01.018 (PMC6355964; doi:10.1016/j.dib.2019.01.018)

Author Conflict Of Interest Declaration

We wish to confirm that there are no conflicts of interest associated with this publication. We confirm that the manuscript has been read and approved by all named authors and there are no other persons who satisfied the criteria for authorship but are not listed. We further confirm that the order of authors listed in the manuscript has been approved by all of us. It is certified that all the financial and material support for this research are clearly identified in the Acknowledgments section of this manuscript.

We confirm that we have given due consideration to the protection of intellectual property associated with this work and that there are no impediments to publication, including the timing of publication, with respect to intellectual property. In so doing, we confirm that we have followed the regulations of our institutions concerning intellectual property.

We understand that the Corresponding Author (Dr. Elsayed Elbeshbishy) is the sole contact for the Editorial process (including Editorial Manager and direct communications with the office). He is responsible for communicating with the other authors about progress, submissions of revisions and final approval of proofs. We confirm that we have provided a current, correct email address which is accessible by the Corresponding Author and which has been configured to accept email from elsayed.elbeshbishy@ryerson.ca.

Signed by all authors as follows:

1- Ahmad Shabir Razavi

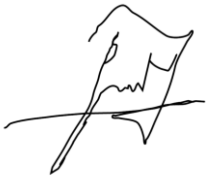

2- Ehssan Hosseini Koupaie

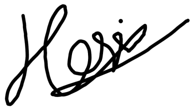

3- Armineh Azizi

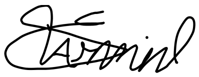

4- Hisham Hafez

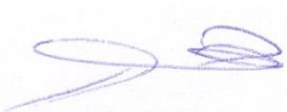

5- Elsayed Elbeshbishy

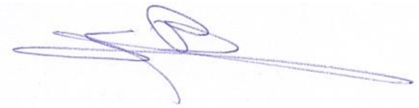

Supplement: Supplementary file 1 — Supplementary material [file mmc1.pdf]
